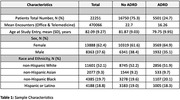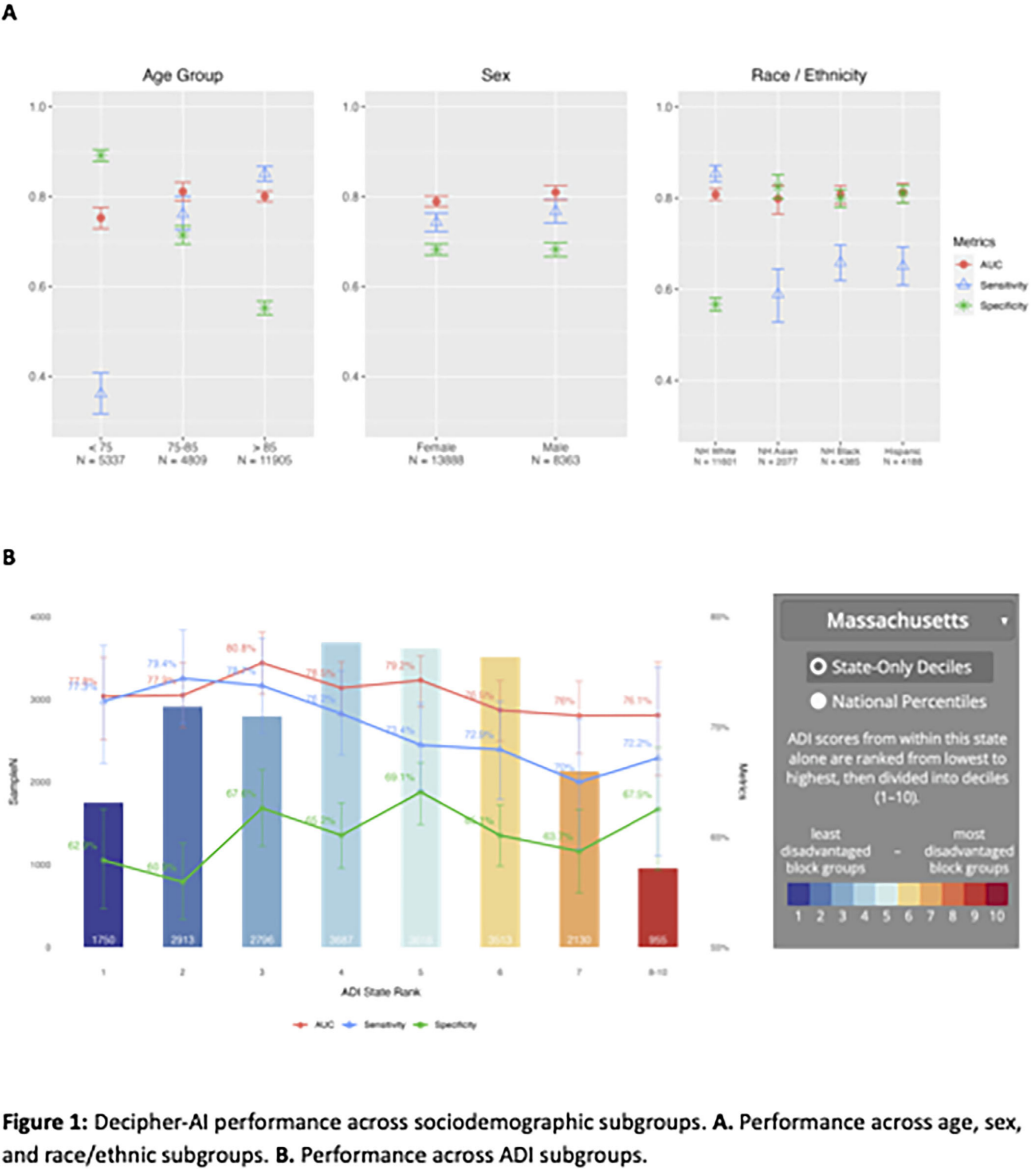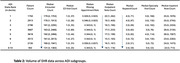# Evaluating Sociodemographic Bias in an Artificial Intelligence Algorithm to Detect Cognitive Impairment in Electronic Health Records

**DOI:** 10.1002/alz.094365

**Published:** 2025-01-09

**Authors:** Colin G. Magdamo, Yignan He, John R Dickson, Tanish Tyagi, M. Brandon Westover, Shibani Mukerji, Christine S Ritchie, Bradley T. T Hyman, Deborah Blacker, Sudeshna Das

**Affiliations:** ^1^ Massachusetts General Hospital, Boston, MA USA

## Abstract

**Background:**

Underdiagnosis of Alzheimer’s disease and related dementias (ADRD) leads to lost opportunities for timely intervention, increased healthcare costs, and underestimation of the true burden of disease. To address this problem, we developed an AI algorithm, Decipher‐AI (DEtection of Cognitive Impairment PHenotypes in EHR), to screen primary care patients for undiagnosed cognitive impairment (CI). We evaluated performance across sociodemographic groups using 3 years of EHR data before the first diagnosis or most recent visit.

**Method:**

Decipher‐AI employs a two‐level hierarchical model, consisting of a large language model (LLM) to generate latent representations from unstructured clinical text and a patient‐level model that combines these representations with structured EHR data to predict the probability of CI (AUC: 0.98, 95% CI: 0.94, 1.00). Decipher‐AI was evaluated on a test set comprising 22,000 Mass General Brigham primary care patients aged 65 years or older. The selection process involved stratified random sampling across distinct racial/ethnic strata to ensure representation of diverse sociodemographic factors (Table 1). Cognitive status labels were determined based on the presence of dementia‐related diagnosis codes, while sociodemographic status was measured using the Area Deprivation Index (ADI).

**Result:**

The AUC on the validation dataset was 0.80 [95% CI: 0.79, 0.81]; sensitivity was 0.75 [0.74, 0.77] and specificity 0.68 [0.67, 0.69], at the threshold of maximum accuracy. There were no significant differences in AUCs across sex or race/ethnic subgroups, but AUC was lower for patients under 75 years. (Figure 1A). Notably, Decipher‐AI exhibited diminished performance in patients from more disadvantaged neighborhoods (Figure 1B). Further analysis revealed that these disparities were associated with fewer encounters, outpatient visits, and notes containing cognition‐related keywords in patients from disadvantaged neighborhoods (Table 2).

**Conclusion:**

Our study highlights the potential of Decipher‐AI for screening undiagnosed cognitive impairment in primary care. Nevertheless, disparities in algorithm performance underscore the importance of addressing sociodemographic inequities in EHR data. In the future, systematic tailored screening in primary care via standardized questionnaires coupled with AI‐assisted chart reviews, has the potential to address the underdiagnosis of ADRD in an equitable manner.